# A Qualitative Exploration of the Economic and Social Effects of Microcredit among People Living with HIV/AIDS in Uganda

**DOI:** 10.1155/2012/318957

**Published:** 2012-06-21

**Authors:** Glenn Wagner, Yashodhara Rana, Sebastian Linnemayr, James Balya, Lydia Buzaalirwa

**Affiliations:** ^1^Health Unit, RAND Corporation, 1776 Main Street, Santa Monica, CA 90407, USA; ^2^Uganda Cares, AIDS Healthcare Foundation, Plot 1 Baker Road, Nakasero, P.O. Box 22914, Kampala, Uganda

## Abstract

HIV medical care, including antiretroviral therapy (ART), is often successful in restoring physical health and functioning. But in developing countries, HIV medical care is often insufficient to achieve social and economic health, and hence innovative economic support programs are much needed. We conducted semistructured interviews with 30 adults receiving ART and microcredit loans operated by Uganda Cares. Using content analysis, we explored the impact of the microcredit loans on the economic, social, and psychological well-being of respondents. Most respondents indicated that the microcredit loans played a positive role in their lives, helped them to keep their children in school and sustain their families, and improved their self-esteem and status in the community. In addition, we also found significant positive knowledge spill-over and network effects in the program with regard to business management and support. However, more than half of the participants indicated experiencing repayment problems either personally or with other group members due to unexpected emergencies and sickness. These findings highlight that microcredit programs have the potential of being an economic support system for HIV clients trying to reestablish their livelihoods, especially in resource-constrained settings, though more research is needed to determine the overall economic viability of such programs.

## 1. Introduction

HIV medical care, especially antiretroviral therapy (ART), suppresses the replication of HIV virus, restores some of the body's ability to fight infections, and consequently helps improve physical health and functioning [1]. But the ultimate goal of HIV care is to enable individuals to achieve social and economic well-being—to work and provide for themselves and their family, keep their children in school, and their households intact [2–4]. In developing countries, where poverty is high and employment opportunities in the formal labor market are few, HIV medical care is often insufficient to achieve this goal, and innovative economic support programs are essential. Microcredit programs have grown tremendously in recent years in Sub-Saharan Africa as they offer the potential to assist individuals in generating income through small enterprises. 

The current predominant model of microcredit provision in developing countries is group-based lending in which a group of clients (typically 6–10) collectively coguarantee loan repayment and access to subsequent loans is dependent on successful repayment by all group members [5]. The group-lending model treats social networks as an asset that substitutes for collateral [6–8]. If a member fails to repay their share, other members apply pressure or offer assistance; otherwise, they must either repay for them or face losing access to future loans. Members are generally required to attend group meetings to discuss issues related to their business and to develop social bonds that help them to provide support, networking, and training to each other [9]. 

By helping households accumulate wealth, microcredit can help reduce their vulnerability to sudden income fluctuations. Even when membership does little to reduce poverty, it may nevertheless reduce household vulnerability to risk and help households to learn to manage risk on their own [10, 11]. Pitt et al. have found that microcredit loans to female borrowers led to increased employment for both male and female household members, more consumption, better nutrition, and increased child schooling [12, 13]. Yet there is concern that these programs may lead to a pattern of “loan recycling” where by loans are acquired to pay-off previous loans, which merely increases the borrowers' debt liability and can lead to increased tensions among household members, domestic violence, and increased dominance over women [14]. Morduch (1999) suggests that although many programs report repayment rates of close to 100%, much of this is disguised by “loan recycling,” while programs that are financially sustainable charge higher interest rates and require collateral, effectively excluding the very poor including many persons living with HIV/AIDS (PLHA) [15]. The operational costs of making small loans results in high interest rates (15–30%), and many microcredit programs require repayment to begin immediately (within one week) [16, 17]. With the growing spotlight on the potential pitfalls of microfinance, it is understandable to question the viability of microcredit loans for PLHA given their added disease-related vulnerability [18]. 

Few studies have explored the effects of microcredit among PLHA. In Thailand, loans to business partnerships involving an HIV-negative and HIV-positive individual not only improved the economic conditions of PLHA but also reduced stigma and discrimination towards PLHA in the community [19]. Barnes (2002) examined the effects of microcredit on entrepreneurs in Zimbabwe and found that among HIV-affected households those that accessed microcredit had more sources for securing income, higher proportion of boys in school, and a savings account in a bank, compared to those that did not access credit [20]. Regarding PLHA on ART, we know of only one qualitative study conducted in Cote d'Ivoire in which the authors found that when PLHA were using appropriate medical treatment, they were capable of engaging in microcredit activities which led to improvements in economic self-sufficiency and provided recipients with psychosocial support [21]. Since ART helps HIV-affected individuals regain physical strength and capacity to work, we reason that microcredit can be a vital economic resource for such individuals to reenter mainstream society after having undergone the debilitating effects of HIV on their income, assets, and household's well-being.

With the growing momentum of microcredit scaleup in sub-Saharan Africa, and in Uganda in particular, a better understanding of the benefits and challenges associated with microcredit for PLHA is needed to enhance the likelihood that such programs can be successful at helping individuals and household overcome the socioeconomic effects of the disease. We conducted semistructured interviews with a sample of ART patients to explore their experiences with microcredit and to understand the impacts of microcredit loans on their economic, social, and psychological well-being. 

## 2. Methods

### 2.1. Description of the Social and Economic Empowerment Program (SEEP)

Uganda Cares is an NGO that operates 13 HIV clinics across Uganda and provides HIV care to over 54,000 patients. Two years ago, Uganda Cares started the SEEP program to offer microcredit loans to their medically stable ART clients. Clients are eligible for the SEEP loans if they are at least 18 years of age, on ART for at least two years and currently earn some form of income. Clients are informed of the program during 4-week mobilization periods in which the program administrator attends the clinic each day and gives a 10-minute presentation about the program to all clients as a group before they start to see the clinicians. Clients who are interested in the program are then directed to attend an orientation meeting at the end of the 4-week mobilization. Clients can only enter the program during the mobilization periods, which have taken place about twice a year. 

The program starts with the orientation meetings followed by an 8-week preloan training period, during which clients meet weekly to learn about program policies and procedures and to receive training related to business management and skills building, as well as instruction regarding their health, nutrition, hygiene, and importance of adherence to ART. Clients also form loan groups of 5-6 members during this period. At the end of the training period, loans are issued to each individual in the group (initial loan amount ranges from Ushs 50,000 to Ushs 300,000). The group then continues to meet weekly to provide their payment to the group's designated treasurer, who then gives it to the program administrator. Each loan has an interest rate of 3% per month and is for 6 months, after which the loan must be fully paid back. Clients who repay their loan on time can then apply for a new, larger loan. To date, approximately 96% of the loan recipients have been able to repay their loans.

### 2.2. Sample

In October 2010, we interviewed 30 adult HIV clients who were receiving ART and microcredit loans from Uganda Cares. Fifteen participants received care at a clinic located in the capital city of Kampala; the other 15 received care at a clinic in Masaka, a town approximately 150 km from Kampala. Seventeen (57%) were male, and 21 (70%) had a spouse or primary partner. All participants were currently on ART and had been receiving microcredit loans from Uganda Cares for at least 6–12 months. 

The participants were a convenience sample, as participants were informed of the study during one of their loan group's weekly meetings. Those who were interested in participating were asked to come to the clinic where they gave informed consent and met with the study interviewers. Participants were paid 15,000 Uganda shillings (~$7 USD) to compensate for transportation costs. The study protocol was reviewed and approved by the Institutional Review Board at RAND and the Uganda National Council for Science and Technology. 

### 2.3. Interview Structure

In-person, semistructured interviews were conducted to elicit information about how clients experienced the microcredit program and the impact of the loan on multiple dimensions. An interview guide was developed to structure the interview. We began the interview by asking participants to describe their work-life before they were HIV-positive until they started receiving microcredit loans. After this “grand tour” question, we used a three-staged framework to probe for more specifics. The three stages or time-periods in our interview protocol were (1) premicrocredit loan, (2) while receiving microcredit loan, and (3) postmicrocredit loan. In the first stage, we asked interviewers to describe how they learned about the program and decided to apply for the loan. We also inquired about their expectations for the loan and how they hoped it would impact their life. In the second stage, we explored the respondent's experience with the loan process, group repayment, and training. In the final stage, we asked respondent's about the impact of the loans on their business and household's economic stability, as well as social and psychological effects. Interviews were conducted in the native language of the participant, which was typically Luganda, or English if they preferred. The interviews were conducted by three graduate students who had received training in qualitative interviewing methodology (including mock interviews and role playing) from G. Wagner and S. Linnemayr. 

### 2.4. Data Analysis

The interviews were digitally recorded with permission from the participant and then translated verbatim into English so that the analyses of the responses are most reflective of the respondent's actual responses and not the interpretation of the researcher or interviewer. To identify themes, we utilized a staged technique described by Lincoln and Guba (1985) and later elaborated on by Ryan and Bernard (2000) [22, 23]. First, we used a text management software *ATLAS.ti*, which helps index primary data. This software allowed us to systematically locate, code, and mark connecting blocks of transcript text that pertained to the major topical domains of interest. After indexing, we extracted all text associated with a particular domain and then two team members sorted quotes pertaining to a domain into several piles based on their thematic similarities. Each thematic category that was identified was given a name and we developed an explicit codebook outlining inclusion and exclusion criteria for each such category. Finally, two team members studied all the quotes and identified which quotes were affiliated with which thematic category under different domains. In the event of a disagreement or confusion, two coders discussed the text in light of the overall interview synopsis and came to a consensus. Finally, we examined the distribution of themes across each thematic code.

## 3. Results

### 3.1. Premicrocredit Loan

#### 3.1.1. Effects of HIV and ART on Work

The ability to work is highly contingent on physical health. Among the 30 interviewees, 83% of the participants described having physical health problems during the time leading up to and around their HIV diagnosis. Participants described their poor health in the context of their work life and how their health negatively impacted their ability to perform their work activities at their normal capacity, a few examples of which are “When I contracted the virus all my work went to a standstill. I tried to provide treatment to myself but all was in vain. Headache was one of my greatest illnesses” (male); and “Time reached when my earnings reduced and I was bed ridden before acquiring treatment at Uganda Cares” (male). In contrast, after having been on ART treatment for a few months, 93% of the participants reported that their physical health had improved significantly, and for most this improved health translated into improved work functioning: “It took me like a period of four months and I began feeling well; regained my lost weight, developed appetite, started doing my work very well, and as I talk now I don't have any problem. I am very ok” (male). The two participants who reported moderate health continued to have bouts of “weakness,” which hampered their work.

After having regained their working capacity, 73% of the participants were doing the same type of work as before their HIV diagnosis. The majority of whom were engaged in microenterprises such as selling fish, second-hand clothes, *matooke* (whipped banana, a Ugandan favorite), or shoes, while others earned income in jobs such as being a farmer butcher, mechanic, or restaurant operator. Of the 27% of the participants who changed their type of work, three had been in salaried positions, one was a home-maker, and the rest were a farmer, fish seller, businessman, and taxi driver. At the time of taking the loan, all were operating their own businesses.

#### 3.1.2. Decision to Enroll in SEEP

When participants were asked to describe the decision processes that led them to apply for the microcredit loan, the most common reasons were to gain capital needed to expand or purchase items for their business (40%), to earn income to provide for their family and pay for basic needs (30%), and to pay for their children's school fees (26%). It is worth noting that less than 25% of the participants had previously accessed loans from conventional microcredit institutions, banks, or less formal village associations; we did not enquire whether these loans took place before or after their HIV diagnosis. 

Nearly 25% of the participants stated that they were interested in applying for SEEP loans as opposed to loans from conventional institutions due to the lack of HIV discrimination, as illustrated in the following testimonies: “I did not think of it because I used to fear other organizations. This was because other organizations had a belief that when they provide loans to people having HIV/AIDS, they would die before paying back their money” (female); and “I was poor and I wanted money to inject in my business. I also had fees problems and even one day I attempted to go for a loan from Faulu Uganda Ltd., but I was denied because I was HIV-positive” (male). 

Similarly, the trust that clients had in Uganda Cares and how the organization provided support and assistance to its clientele (e.g., providing free ART to patients and encouraging them to adhere to their medication regimens) were cited by participants as a reason for their decision to apply for a loan. Other reasons cited by participants were the social aspects of the program, such as the sharing of business-related ideas, development of social support networks for income generation, and simply getting to know other HIV-infected individuals on a more personal level.

### 3.2. During the Microcredit Loan

#### 3.2.1. Experience with Preloan Training

While describing their impressions of SEEP's training program, all of the 28 participants who responded to this question found the training beneficial. Among the multiple reasons cited by each participant, 90% indicated that the various training materials on business management were helpful for specific business-related activities such as book keeping, how to best grow vegetables, and how to deposit money into a bank, as well as general advice about how to improve one's business and the importance of establishing savings. 28% of the participants also described social-related benefits from the training content such as how to overcome HIV stigma and live positively, and to build strong relationships. Benefits for nutrition, disease prevention (e.g., malaria) and proper hygiene were also noted.

While there were several benefits that participants received from the training program, they also noted several shortcomings especially related to trainers and training content. With regards to the trainers and administration of the training, respondents called for the trainers to be more reliable in attending and showing up on time for scheduled training sessions, to limit frequent switches in who administers the training, and avoid abruptly stopping or interrupting the schedule of training. With regard to training content, respondents wanted more training specific to business management skills such as book keeping and accounting, accountability for inventory and funds, business plan development, and training for specific income-generating activities such as sewing and poultry farming. Finally, a few responses were related to the structure of the training program, indicating that the training period was too long (8 weeks) and too frequent (weekly), the latter of which was in turn related to high transportation costs for some.

#### 3.2.2. Experience with Loan Repayment and Group Responsibility

In the case of loan repayment, we focused on the challenges that participants faced with repayment but did not document whether participants had actually missed payments during the 6-month loan cycle. For this reason, while we are unable to discern how many participants were successful at the end of their respective loan cycles, we are able to provide a range of challenges encountered. While 40% respondents reported no difficulties in making their loan payments, all the other participants reported having problems. The most common challenge cited by 30% of the participants was the requirement for weekly payments, which seemed to be particularly problematic for those with seasonal jobs: “Some of us our businesses are seasonal whereby you might have great sales for one season and the other season poor sales like now; us who sell at the park yard market, when it rains we suffer a lot and at times we are forced to close” (female). Similarly, interruptions to income generation and business operation caused by unexpected emergencies or circumstances, and sickness, were the other challenges to loan repayment that were reported by multiple respondents.

With regards to their feelings of being in groups, participants indicated having experiences that were often positive and beneficial. Of the 28 participants who described their perceptions about their groups, roughly 70% indicated that they liked being part of a group because the interactions between group members went beyond loan repayment into more supportive and social interaction. Similarly, 70% of the participants also indicated that their group members helped each other in other ways such as collecting funds to provide for a member who had lost a loved one or for sickness, emergency, or social events; buying commodities from each other; lending money to each other; delivering ART medication to a member who could not make it to the clinic.

While a majority of the participants liked being in groups, they also noted several shortcomings. First and most important of all, 67% of the participants stated that they suffered from other group members not making payments on time. In the case of a missed payment, other group members would pay for the defaulter while the defaulter was expected to pay at a later date. Once again, we need to caveat this finding with the fact that we did not document whether other group members who had missed their payments defaulted at the end of their loan cycles. Respondents also noted that some members did not attend meetings on time or just sent their payments with others instead of attending themselves; others are perceived to be only interested in making their payments and not interacting with or supporting the work of others. 

### 3.3. Postmicrocredit Loan

#### 3.3.1. Loan Impact on Business and Income

At the time of the interviews, all participants indicated that they were engaged in microenterprises. Roughly 75% of the participants earned income by selling things such as fish or other foods, shoes, and used clothing, but two participant also reported having a shop and selling retail merchandise. Other jobs included restaurant operator, *boda boda* (transporting people on a motorcycle), taxi driver, mechanic, butcher, and breeding chicken or other animal husbandry. When asked to describe how well these businesses were currently functioning, of the 26 participants who responded, 38% described their business as performing well, using terms such as “running smoothly” and “it has expanded a bit,” but 27% stated that their business was “doing very badly” or “not going well”; the other 35% gave a more neutral response such as “it keeps changing” or “it's 50/50, sometimes good, sometimes not so good.” This range in business performance was present among both male and female respondents.

Even though more than 25% of the participants' businesses were not doing well, 90% of the respondents deemed the microcredit loan to have played a positive role, with many indicating that the loans enabled them to expand their business, increase income, and taught them how to save. One male participant exclaimed: “This loan has helped me to stay in touch with my customers. But before the loan, they had shifted to other shops because I did not have what they wanted. Like if a customer comes to buy a razor blade, he realized that I have also stocked rice and beer and this makes such a customer to come back for those items.” Only 6% of the respondents described the loans as having a negative impact, indicating that the loans were not enough or lost which resulted in stress: “That loan caused stress in my life because I lost the money. But if they had not stolen that money, it would be a good loan because it would have helped me to do all the work I had prepared. I would have bought a vehicle that I would have used for transporting the items” (male).

When asked if their business would have been sustainable without the loans, 50% of the participants believed that their business would be sustainable without the loans but these beliefs were often qualified with statements such as “but with difficulty,” “but at a lower, smaller or slower rate,” “but we would have struggled,” and “but it wouldn't have been as vibrant as it is now”—revealing the perceived benefits of the loans. Several others felt that they would have struggled to sustain their business without the loans, indicating that they needed capital to continue their businesses. One-fifth of the respondents indicated that they could access funding and loans from other sources if needed. In a separate question, 60% of the respondents indicated that they had no other options then SEEP for acquiring loans, citing complex bureaucracy, required collateral, and the fear of burdening their family with unpaid debt if they passed away. In addition, several clients still felt that other institutions would discriminate against HIV-infected individuals and thus not provide loans: “Because other banks have a lot of requirements before you are given a loan like security, guarantors and worst of all they were discriminating us who are HIV positive” (female).

#### 3.3.2. Social and Psychological Impact of the Loans

Consistent with SEEP policy that loan funds should be applied mostly to business operations and expenses, of the 30 respondents, 83% of the respondents indicated that their loan was applied towards expanding their business. Yet likewise, nearly all respondents reported that they also used loan funds for nonbusiness purposes. Most participants indicated that the loans were at least partially used to pay for children's school fees, while others used the funds to pay for household necessities and basics for their family, food, clothing, and rent.

Since we expected the social and economic benefits of the loan to impact self-perception, we also enquired from participants how they felt about themselves. Most respondents described positive psychological effects of the loans, indicating that they felt “happy” or “hopeful,” or that they had no fears or anxiety regarding loan repayment. Others referred to their self-esteem and spoke of feeling “confident,” “less shy,” “proud” or “admired.”   Some suggested that the loans and related income generation and business performance contributed to reduced stigma both internally and from others: “Before getting this loan things were not easy, but when I got my first loan and my business moved on smoothly I came to believe that being with HIV doesn't mean that life has ended” (male).

Further indications of lowered stigma were evident when participants spoke about their status in the community. Of the 23 participants who responded to this question, 21% of the respondents stated that there was no change, while only 9% of the respondents indicated a negative change fearing that their community perceived them to be “showing off” or accused them of being a thief because of their increased income. However, 65% of the respondents described receiving greater support and respect from those in the community, and others reported an expanded social network and increased social invitations and popularity. While most of our findings revealed no evidence of any gender differences, this is the one area where we did see a noticeable difference in the experience of male and female clients. While a few women reported increased support and respect from the community, most indicated no effect or change. In contrast, the majority of male respondents described a perceived increase in respect and support from members of the community, which is implicit in the following testimony: “Yes my status has changed very much. Well these days whenever problems come up around my area I am always taken as the best adviser. And also whenever there are occasions I am always invited, and in my village I was appointed on the village council. Whenever there are parties in my village I am seated on the high table. Yes, because as I say now I am the rich person in my village” (male).

While many respondents described beneficial psychological effects of the loans, some also reported negative effects. Several described being stressed out and more anxious because of the responsibility to repay the loans. Others spoke of stress from their business being much busier and feeling the pressure to work harder. As one of the female respondent notes: “This loan has not changed my life for the better because it makes me too busy. When it's a market day I can't stay at home or rest I have to go by all means because I have to pay a loan” (female).

### 3.4. Suggestions for How to Improve SEEP

Finally, participants were asked about areas in which they thought SEEP could improve; 70% of the 30 respondents requested for the repayment schedule to be changed from weekly to something less frequent (most suggested biweekly or monthly). Other common requested changes were options of taking out larger loans, lowering the interest rate, more varied and frequent training opportunities (related to business management as well as specific business skills), and the option of taking out a loan individually rather than as a group. Some respondents indicated an interest in having SEEP expand their area of assistance beyond loans and business support to areas such as assistance with children (e.g., paying school fees, supporting orphans), improving water supply, helping to build homes, and helping clients establish group-operated income generating projects.

## 4. Discussion

These detailed discussions with PLHA on ART who are currently receiving microcredit reveal considerable diversity in the benefits and challenges associated with microcredit in this population. First, we believe that SEEP's microcredit loan is correcting an important market failure by providing loans to those who need it yet feel shunned by both the formal and informal credit market systems. Similar to previous studies, we found that after being on ART most participants had regained physical strength and were able to resume work [24]. Yet, the consequences of HIV on the socioeconomic well-being of affected individuals render many PLHA in need of economic support even after receiving HIV medical care. Microfinance is one type of such economic support, but only when it is offered through AIDS service organizations do PLHA generally find microcredit loans to be accessible. More than half of the participants in our study were interested in applying for microcredit loans because they wanted to expand their business and/or they desired to work. Yet, roughly two thirds of them had never accessed loans from other formal and informal institutions before enrolling in SEEP. Likewise, at the time of the interviews, the same proportion of participants still felt that they could not access other sources of funding, citing complex bureaucratic systems, requirement for collateral and fear of confiscation of property. Among these reasons, the most frequently cited was the perception that other institutions discriminated against their HIV status. 

Second, our data reveal mixed evidence with regards to the economic benefits of microcredit loans for PLHA. More than half of the respondents indicated that their businesses were performing well or neutral, and the vast majority indicated the loan had played an overall positive role in their lives. Yet many respondents stated having had repayment problems either personally or with other group members. Despite being on ART and having access to microcredit loans, PLHA continue to face seasonal income shocks that make the weekly payment system problematic. In addition, many participants indicated that they had to divert money to pay for school fees and occasionally for treatment due to bouts of sickness. To fully discern the economic feasibility of the loans, more systematic, quantitative data are needed regarding loan repayment and default rates, as well as business income, profits, and debt. 

Third, although the economic impact of the loans may be mixed, there is clear evidence that the loans have positive social and psychological effects. When we asked respondents about their ability to take care of their families, most respondents indicated that they were able to send their children to school and keep their families intact. More than two-thirds of the respondents felt good about themselves though some respondents mentioned that, while they were happier overall and confident, they were also stressed about not meeting their weekly payments. Equally important is the fact that more than half of the participants felt that their status had improved in their respective communities. These findings are in agreement with social cognitive theory, both of which posit that a positive change in an individual's economic stability can influence a person's self-efficacy and thereby have a critical influence on behavior and performance related not only to income generating activities but also social outcomes [25–28]. The findings from this study suggest that, when physical health and functioning are largely restored, microcredit loans help to bolster confidence, perseverance and motivation for expanding one's business and improving overall economic well-being. 

Fourth, we also found the microcredit loans to have positive spill-over effects on other dimensions. All participants indicated that they benefited from having had the opportunity to learn about book keeping and other business management skills, importance of savings, and procedures to follow when paying back loans. They also indicated that they appreciated learning about nonbusiness-related aspects of how to improve their health and quality of life, including how to live positively with HIV. The positive impact of the training program was also evident when we asked participants to list the main benefits of the SEEP program, as the fourth most cited reason was the training program. Furthermore, more than half of the participants expressed the desire to receive more training. For these reasons, we believe that microcredit programs should place more of an emphasis on developing innovative approaches to offering training and education programs to their client base, in the realm of business management and operations, as well as other dimensions of overall health and well-being.

Fifth, while the microcredit literature has mostly looked at the issue of networks in terms of accountability, our qualitative data show that such networks also function as strong support structures. In our analysis, more than half of the participants mentioned that they liked being part of a group. Many respondents reported that group members assisted each other to help make repayments and shared ideas related to business. They also mentioned that each group established a common fund and members pooled a certain percentage of their money in this fund to help other members. In the case of PLHA, we believe that such networks can play a crucial role in overcoming stigma and engaging in positive thinking and living. While we did not specifically probe for the impact of networks on stigma and self-perception, some participants mentioned that the program helped them build “networks of new friends” and overcome stigma, evident in the following testimony, “Our life situation is not easy. But the moment you meet the person who is also infected you gain some strength and become consoled. It has therefore helped us to fight against stigma.” 

With this study's small convenience sample, these findings are not representative of all PLHA receiving ART and microcredit loans. Nonetheless, the study interviews provide important insights into the effects of microcredit on the well-being of clients in the lower economic stratum of Ugandan society. The interview data highlight substantial economic, social, and psychological benefits with microcredit. By providing respondents with the ability to expand their businesses, keep their children in school, and maintain the livelihood of their families, microcredit loans play a positive role in the lives of PLHA. These loans not only improved the self-perception of PLHA but also uplifted their status in their communities. It also provided them with opportunities to learn and network with other PLHA. We are currently conducting a prospective longitudinal cohort study to quantitatively evaluate the economic, social, and sexual impacts of microcredit loans among SEEP clients. We believe the results of our upcoming study will help us evaluate the overall viability of microcredit loans as a critical policy lever for improving the socioeconomic health and well-being of PLHA in resource-constrained settings. 
